# Emotion regulation strategies in bulimia nervosa: an experimental investigation of mindfulness, self-compassion, and cognitive restructuring

**DOI:** 10.1186/s40479-020-00129-3

**Published:** 2020-07-03

**Authors:** Johannes Baltasar Hessler-Kaufmann, Julia Heese, Matthias Berking, Ulrich Voderholzer, Alice Diedrich

**Affiliations:** 1 Schoen Clinic Roseneck, Am Roseneck 6, 83209 Prien am Chiemsee, Germany; 2 Department of Psychiatry and Psychotherapy, University Hospital, LMU Munich, Munich, Germany; 3 Department of Psychology and Sport Sciences, University of Erlangen-Nuremberg, Erlangen, Germany; 4 Department of Psychiatry and Psychotherapy, University Medical Center Freiburg, Freiburg, Germany

**Keywords:** Bulimia nervosa, ER, Self-compassion, Mindfulness, Cognitive restructuring, Inpatient

## Abstract

**Background:**

While improving emotion regulation (ER) is a central goal in the therapy of bulimia nervosa (BN), there is no experimental evidence on the efficacy of different ER strategies. (1) We hypothesized that mindfulness as well as self-compassion as contextual strategies and cognitive restructuring as classical cognitive behavioral strategy would outperform waiting in improving emotional and eating disorder related outcomes after an unpleasant mood induction. Further, we explored (2) whether contextual strategies outperformed cognitive restructuring and (3) whether comorbid mental disorders and previous treatment for BN influenced the efficacy of contextual ER strategies compared to cognitive restructuring.

**Methods:**

Within their first 2 weeks of treatment, inpatients with BN were instructed to utilize mindfulness, self-compassion, and cognitive restructuring or to wait after a pre-induced sadness in a permuted repeated measures design. Patients further rated different emotional and cognitive outcomes on a visual analogue scale at baseline, and before and after each ER strategy. Multiple linear regression analyses were employed to compare (1) the active conditions to waiting, (2) the contextual strategies with cognitive restructuring, and (3) the latter analysis again, but separated according to comorbidity and previous treatment.

**Results:**

Forty-eight female inpatients with BN (mean age = 26.44 years, *SD* = 6.64) completed the study. (1) Contextual ER strategies were more efficacious than waiting for eating disorder symptoms. Cognitive restructuring did not differ from waiting for any outcome. (2) Contextual strategies were more efficacious than cognitive restructuring for emotional outcomes. (3) Self-compassion was more efficacious than cognitive restructuring in patients with comorbid mental disorders and previous treatment in increasing control over the present feeling.

**Conclusions:**

Contextual strategies, especially self-compassion, seem more efficacious than waiting and cognitive restructuring in improving short-term ER in patients with BN in an experimental setting.

## Background

Given the transdiagnostic nature of emotion regulation (ER) deficits in mental disorders [1], bulimia nervosa (BN) has also been associated with a range of related problems. Compared to healthy controls, patients with BN experience their emotions more intensely, report a lower acceptance of aversive emotional states [2], show a reduced ability to regulate unpleasant emotions [3], and indicate greater problems in identifying emotions as well as less awareness of their internal emotional states [4].

Drawing on a transdiagnostic, functional understanding of symptoms of mental disorders, binging and purging behaviors, which are at the core of BN, are considered maladaptive coping strategies, which may offer a distraction from aversive physiological-emotional arousal and provide brief episodes of pleasant feelings and relieve [5–7]. Experimental studies showed a stronger urge to hunger and binge, as well as increased food intake after an aversive mood induction in individuals with binge eating disorder and BN [8–12]. As abstinence from binging and purging is a primary aim in the therapy of BN, the assumed regulatory mechanism of these behaviors emphasizes that deficits in ER should be focused in therapy. While rapid improvements in ER predicted better short- and long-term outcomes [13, 14] and ER was shown to mediate the effect of treatment on outcome [15], it has hardly been investigated, which ER strategies are actually helpful for patients with BN.

Many patients with eating disorders (EDs) remain symptomatic or only show partial improvement after cognitive behavioral therapy (CBT) [16, 17]. Given that central aspects of ER dysfunction in EDs include lack of emotional awareness, clarity, acceptance, reappraisal, as well as problem solving [18], and only the latter two being directly targeted by classic CBT interventions, such as cognitive reappraisal, that focus on changing cognitions and emotions, the therapy of EDs might benefit from incorporating third-wave contextual ER strategies [19]. Rather than trying to alter mental phenomena, contextual methods like Mindfulness Self Compassion Training (MSC [20]) and Acceptance and Commitment Therapy (ACT [21]) focus on the contexts, in which emotions and thoughts arise, and how we relate to them. When we, for example, experience mental pain related to daily phenomena, contextual approaches would instruct us to notice these feeling, open ourselves for the experience, and take on a complaisant stance on this experience. Fighting, that is, trying to change this experience, and possibly failing to do so, would be considered adding self-made pain to the anyways naturally occurring pain.

Mindfulness implies being aware of the present moment and paying purposeful attention to own actions, thoughts, emotions, and physical states without judging them [22]. Given the well established negative association between mindfulness and ED symptoms [23–27], mindfulness-based methods gained increased attention for the treatment of ED and other disorders. In BN, mindfulness might mean noticing unpleasant emotional states and the associated impulses to binge and purge, yet, to have the liberty to decide how to act upon these phenomena [26, 28, 29]. While a pilot study with a small group of women with BN a mindfulness-based treatment program reduced emotional distress [30], there is still a lack of experimental data regarding the efficacy of mindfulness as an ER strategy in BN.

Self-compassion was described as “a strong and warm feeling of empathy towards oneself in distress that is associated with the desire to help oneself” [31]. Hence, self-compassion directly opposes self-criticism, which is strongly associated with ED symptoms, including body dissatisfaction, drive for thinness, body preoccupation and external shame in student [32, 33] and clinical samples [34–36]. While low self-compassion was associated with poorer treatment response in ED [37], introducing Compassion Focused Therapy (CFT) to standard therapy program for EDs, patients with BN showed a significant improvement in their ED pathology [38]. As with mindfulness, however, there are, to our knowledge, no experimental data for the efficacy of self-compassion in persons with BN until now.

Given the need to further improve treatment for BN and the lack of experimental data on contextual approaches to ER in EDs, the aim of the present study was to compare the effect of cognitive restructuring, mindfulness, and self-compassion on outcomes related to emotions (sadness, acceptance for feeling, tolerance for feeling, perceived control over feeling) and EDs (urge to eat, urge to purge, acceptance for shape and weight, feeling valuable) in inpatients with BN. (1) We hypothesized that the active strategies (restructuring, mindfulness and self-compassion) were all more efficacious than a waiting condition in reducing sadness and improving further symptoms. (2) In exploratory analyses, we investigated whether contextual ER strategies (mindfulness and self-compassion) were more efficacious than cognitive restructuring as traditional CBT intervention and (3) whether comorbid mental disorders and previous treatment, as respective proxies for psychopathological load and experience with applying psychotherapeutic strategies, influenced the efficacy of the contextual ER strategies.

## Methods

### Participants

Participants were 48 inpatients of the Schoen Clinic Roseneck in Rosenheim, Germany, which is specialized in the treatment of EDs. The experiment was conducted within the first 2 weeks after their admission to inpatient treatment. Patients were included if they were female, at least 18 years old, fluent in German, and met the DSM-IV criteria for BN. Exclusion criteria were organic brain disorders and a high risk of suicide. Participants with comorbid disorders in addition to BN were included except for patients with comorbid psychosis, bipolar disorder, substance abuse, or addiction. We included 48 patients to be able to detect moderate effects with sufficient power in the planned design.

### Procedure

All patients, who reported symptoms of bulimia at admission and met the inclusion criteria, received an information sheet about the study and were asked to participate. All participants, who provided informed consent, were invited for an interview. In this interview, diagnoses of BN and comorbid disorders were assessed by conducting the Structured Clinical Interview for DSM–IV Axis I and II Disorders (SCID; German version: [39]). The study was approved by the local ethic committee of the Ludwig-Maximilian-University Munich.

Up to 7 days after the interview, the experiment took place in a laboratory in the Schoen Clinic Roseneck in Rosenheim. Participants sat at a desk in front of an *ASUS* computer. Presentation software (Neurobehavioral system, Albany, CA) was used for the experiment. After explaining and starting the experiment, the investigator left the room. During the experiment, there were four unpleasant mood induction phases, each followed by a phase with a respective ER strategy. Participants rated their sadness before and after each mood induction and before and after each strategy. In the mood induction phase, patients were instructed to listen to “Adagio in G minor” by Tomaso Giovanni Albinoni while going through a modified Velten mood induction procedure [40], which entails the display of several negative ED related statements (e.g. “I am too fat”) on the screen. The efficacy of the Velten procedure [41, 42] and mood-suggestive music alone, as well as the combination of both methods have been established in previous studies [42].

After each mood induction, the participants spent 5 min conducting one of the four ER strategies. Each strategy started with the following instruction: “The speaker will teach you a strategy to regulate your mood. You can close your eyes if you like. Please click to go on”. By pushing the button, the participants started with the respective strategy. All participants completed all ER strategies. In order to control for sequence effects, complete counterbalancing was applied by permuting all possible sequences (4! = 24).

The cognitive restructuring instruction was as follows: “Read the statements very carefully. Choose one of the statements you can identify yourself with. Which one you think about more often and which has a negative impact on your mood. Take the time. If you found a statement, click on it. If you want to, you can change the statement. Read it closely again and think about it. What arguments validate this statement? Collect arguments for yourself. What arguments invalidate this statement? Do you recall situations that support the correctness of the statement? Can you remember situations that question the validity of the statement? What are the consequences of thinking this way? What are the advantages and disadvantages of this thought? Think carefully about the consequences of your statement. How do you feel if you think like that? Does this thought help you to feel the way you want? And how does it influence your behavior when you think like that? Does this thought help you to behave as you like? Now, try to formulate a more positive, helpful statement based on the selected statement. Formulate the old, selected statement in a more positive one. Just try out several variants until you find one that feels good. If you want to, repeat this new positive statement a few times, change it again, if necessary, until you realize that you are in a more positive mood. Try to notice even small improvements.

The mindfulness instruction was as follows: “Please focus your attention on your breathing. Just try to observe your breath without controlling it. Tell yourself “in” when breathing in, “out” when breathing out, and try to focus on the feeling of the breath as fully as possible. If you notice that you digress, make a brief mental note like “drifted” or “thought” and focus on your breath again. If you begin to get angry that you’re digressing again and again, make a mental note like “Ah, there’s anger” or just “anger” and focus your attention again on your breathing. If there is the right moment for you, draw your attention away from your breathing and try to feel exactly what kind of feelings and moods are present right now. How do you feel? Try to briefly describe the feelings you are experiencing. Perhaps you can also rate the intensity of your feelings on a scale of 0-10, e.g. “Ah, there is sadness, and it’s just about 8, etc.” It is only about perceiving and labelling the feelings. It is not necessary to change them. Perhaps you can look more closely in which body sensations this feeling is reflected. Which impulses and goals are connected with this feeling? And what are your thoughts? In the end, go back to your feelings and check if you have really perceived and labeled everything that is currently activated. And then you slowly come back at your pace from the relaxation and the “mode of the appreciation-free perception” by maybe stretching for a short time. Breathe in deeply and then open your eyes again.”

The self-compassion instruction was as follows: “Try to feel very clearly which feelings have been activated by these statements. Try to watch yourself from an outsider’s view, from the perspective of an engaging, friendly viewer. Imagine what you look like, sitting in front of the computer. Perhaps you can recognize from the outside what feeling stresses you at the moment. Then try to let the warm and powerful feeling of compassion arise within yourself. The special feeling of sympathy, that is associated with the desire to help you. If you recognize this feeling, you can begin to approach yourself and signalize that you are there for yourself, that it is ok to feel like that. Maybe you can say to yourself: “It is ok how you feel. This is a difficult situation. But I am there for you. l will help you.” In the next step, you can start to encourage yourself: “You can do this. You can pull yourself out of this mood again. You have done so much, you can accomplish it now.” If you want, you can also put a hand on your shoulder in your imagination, or you can hug yourself for support. Then try to cheer yourself up by giving yourself a friendly smile. And meanwhile you can check if there are other things you want to tell yourself. Take your time for it. And then you can say goodbye to yourself in this difficult situation. Remind yourself that this is not a farewell forever, but that you can always come back to yourself. Perhaps there is something else you want to tell yourself as a farewell.”

The computer screen showed the following instruction for the waiting condition: “There will now be a short break. Please just remain seated calmly during this time. The program will signal the end of the break to you.”

The outcome variables were assessed at baseline, before and after each mood induction and each strategy and had to be answered on a computer based visual analogue scale (VAS) with the anchors “not at all” (= 0) and “completely” (= 100). The respective questions were: “How sad do you feel?”, “How well are you able to accept this feeling as present?”, “How well are you able to tolerate this feeling?”, “How well are you able to control this feeling?”, “How strong is your urge to eat right now?”, “How strong is your urge to purge right now?”, “How well are you able to accept your shape and weight right now?”, and “How valuable to you feel right now?”

After completing all of the four negative mood inductions and ER strategies, the participants received a pleasant mood induction procedure to counter unpleasant emotions. To induce a positive mood, “An der schönen blauen Donau” by Johann Strauss was played as well as positive ED-related statements (e.g., “I like parts of my body”, “Nobody is perfect”) were shown on the computer-screen.

After the experiment, the participants received a short, self-developed questionnaire to assess the efficacy of the four strategies. They had to answer what they were doing in the waiting condition, if the laboratory setting made them insecure, and how they liked the speaker’s voice. To check whether patients actually followed the instructions and applied the strategies, they were asked if they were able to apply the respective strategies and how much the strategies improved their mood. The participants also rated to what extent they had difficulties with the strategies. Answers were rated on a 5-point scale ranging from 1 (not at all) to 5 (completely).

At the end, all patients received a debriefing in which the current mood was assessed and received a 10 Euro Amazon voucher as compensation. The participants were asked how they felt and if they experienced any bulimic symptoms or impulses. No participant needed a crisis intervention after the experiment.

### Statistical analyses

As preliminary analyses, we examined if there were differences in the effects of mood induction depending on the previously applied strategy. Therefore, we conducted paired-sample *t*-tests as well as a repeated measures analysis of variance (RM-ANOVA) with sadness changes as within subject factor. We conducted four univariate analyses of covariance (ANCOVAs) to assess possible order effects. We calculated one ANCOVA for each strategy with position number in the paradigm as the fixed factor, post-strategy rating of the mood as the dependent variable and pre-strategy rating as the covariate.

The hypothesis that all of the strategies (cognitive restructuring, mindfulness, self-compassion) were more efficacious than the waiting condition was examined with several multiple linear regression models. The strategies were dummy-coded to allow for comparison with the waiting condition (d1: mindfulness vs. waiting; d2: self-compassion vs. waiting; d3: cognitive restructuring vs. waiting) and all models were adjusted for baseline and pre-strategy values of the respective dependent variables. To explore whether third-wave strategies might work better than cognitive reappraisal similar multiple linear regression models with respective dummy codes (d1: mindfulness vs. cognitive restructuring; d2: self-compassion vs. cognitive restructuring) were calculated. The influence of comorbid mental disorders and previous treatment for BN on the efficacy of the contextual ER strategies was explored by conducting the above regression analyses separately according to the levels of the respective variables (no comorbidity vs. comorbidity; no previous treatment vs. previous treatment). While this method may include a loss of statistical power, lack of more detailed information about the number previous treatments and a very skewed distribution of the number of comorbid diagnoses precluded any other method.

To correct for multiple testing, the critical value for α was set at .05/4 = .0125 for each group of outcome (emotions, ED-symptoms) within each analysis (active strategies vs. waiting, third-wave strategies vs. cognitive restructuring, influence of previous treatment, influence of comorbid mental disorders). Statistical analyses were performed with SPSS 21 for Windows and Macintosh.

## Results

### Preliminary analyses

Mean age of the patients was 26.44 years (*SD* = 6.64, range 18–47). The mean duration of the ED was 9.66 years (*SD* = 6.89, range 1–33) and mean body mass index at admission was 22.24 (*SD* = 4.23, range 17.51–39.14). Thirty-five (72.9%) reported to having received previous in- or outpatient treatment, 32 (66.7%) had at least one comorbid mental disorder at admission with depressive disorders (20, 41.7%), personality disorders (15, 31.3%), and social phobia (12, 25.0%) being the most common.

A repeated measures ANOVA with the mood induction change scores as dependent variable determined that mean sadness changes before the respective strategies showed no statistically significant difference, *F* (3,141) = 2.48, *p* = .063, partial *η*^*2*^ = .05. Given the borderline statistical significance of the test, paired-samples *t*-tests were calculated in order to be able to map possible differences in mood induction in detail. These tests revealed that the average sadness changed during the mood induction from 52.58 (*SD* = 29.07) to 60.10 (*SD* = 31.71) prior to cognitive restructuring (*t* (1,47) = 1.78, *p* = .082, Cohens *d* = .083), from 51.75 (*SD* = 26.72) to 57.58 (*SD* = 29.14) prior to mindfulness (*t* (1,47) = 2.33, *p* = .024, Cohens *d* = .209), from 48.90 (*SD* = 30.04) to 62.60 (*SD* = 27.79) prior to self-compassion (*t* (1,47) = 3.87, *p* < .001, Cohens *d* = .473), and from 46.44 (*SD* = 28.10) to 62.77 (*SD* = 27.77) prior to waiting (*t* (1,47) = 4.74, *p* < .001, Cohens *d* = .585). While *p*-values indicate that the mood induction might have been similarly effective in all four strategies, values of Cohen’s *d* for the *t*-tests suggest that the mood induction might have had effects of different sizes, ranging from no effect before cognitive restructuring to a moderate effect before waiting.

There was no statistically significant effect in the post-strategy rating for any of the four conditions depending on their position, indicating that we were able to control possible order effects by applying all possible sequence permutations.

Adjusted multiple linear regression models revealed that mindfulness was superior to waiting with regard to increasing acceptance for shape and weight (Table 1). Self-compassion was a better ER strategy than waiting with regard to increasing acceptance for shape and weight and the feeling of being valuable. Cognitive restructuring did not differ from waiting for any of the examined variables. Pre-strategy values of the outcome variable were the strongest predictors in all models.

**Table 1:** Adjusted efficacy of active emotion regulation strategies compared to waiting in inpatients with bulimia nervosa: results of the multiple linear regression analyses

Variables	*b* (95% CI)	*p*	*β*
DV: sadness			
Mindfulness	−6.00 (−14.42; 2.42)	.161	−.10.
Self-compassion	−8.66 (−17.05; −0.28)	.043	−0.14
Cognitive restructuring	−5.13 (−13.52; 3.26)	.230	−0.08
DV: acceptance for feeling			
Mindfulness	6.22 (−1.65; 14.09)	.120	0.12
Self-compassion	3.68 (−4.28; 11.64)	.362	0.07
Cognitive restructuring	−4.37 (−12.65; 3.91)	.299	−0.08
DV: tolerance for feeling			
Mindfulness	5.85 (−2.33; 14.03)	.160	0.10
Self-compassion	8.01 (−0.21; 16.24)	.056	0.14
Cognitive restructuring	−2.91 (−11.53; 5.72)	.507	−0.05
DV: control feeling			
Mindfulness	−1.40 (−8.43; 5.64)	.696	−0.03
Self-compassion	7.09 (0.02; 15.16)	.049	0.13
Cognitive restructuring	−5.45 (12.85; 1.95)	.148	−0.10
DV: urge to eat			
Mindfulness	−4.84 (−11.49; 1.80)	.152	−0.07
Self-compassion	−0.41 (−7.10; 6.29)	.905	−0.01
Cognitive restructuring	2.08 (−4.65; 8.82)	.542	0.03
DV: urge to purge			
Mindfulness	−5.75 (−12.70; 1.20)	.104	−0.08
Self-compassion	−4.27 (−11.28; 2.74)	.231	−0.06
Cognitive restructuring	1.40 (−5.78; 8.58)	.700	0.02
DV: acceptance for shape and weight			
**Mindfulness**	**8.73 (2.45; 15.00)**	**.007**	**0.17**
**Self-compassion**	**8.34 (2.04; 14.65)**	**.010**	**0.16**
Cognitive restructuring	4.52 (−1.98; 11.02)	.172	0.09
DV: feeling valuable			
Mindfulness	7.41 (0.78; 14.05)	.029	0.14
**Self-compassion**	**9.94 (3.25; 16.63)**	**.004**	**0.19**
Cognitive restructuring	1.49 (−5.63; 8.61)	.680	0.03

Independent variables dummy-coded for comparison with the waiting condition and adjusted for baseline and pre-strategy values of the dependent variable

*95% CI* 95% confidence interval, *DV* dependent variable

Effects with *p* < .0125 in **boldface**

Adjusted multiple linear regression models suggested that third-wave ER strategies worked better than cognitive restructuring as classic CBT intervention (Table 2). Mindfulness outperformed cognitive restructuring with regard to increasing acceptance for the current and self-compassion was superior in increasing perceived control over the feeling. Pre-strategy values of the outcome variable were the strongest predictors in all models.

**Table 2:** Adjusted efficacy of third-wave emotion regulation strategies compared to cognitive restructuring in inpatients with bulimia nervosa: results of the multiple linear regression analyses

Variables	*b* (95% CI)	*p*	*β*
DV: sadness			
Mindfulness^a^	−1.04 (−9.58; 7.50)	.810	− 0.02
Self-compassion^a^	−3.37 (− 11.91; 5.18)	.437	− 0.06
DV: acceptance for feeling			
**Mindfulness**^**a**^	**10.96 (2.79; 19.12)**	**.009**	**0.22**
Self-compassion^a^	8.34 (0.22; 16.49)	.044	0.17
DV: tolerance for feeling			
Mindfulness^a^	8.27 (−0.67; 17.21)	.070	0.16
Self-compassion^a^	10.41 (1.40; 19.41)	.024	0.19
DV: control feeling			
Mindfulness^a^	3.29 (−3.78; 10.35)	.359	0.07
**Self-compassion**^**a**^	**11.65 (4.48; 18.82)**	**.002**	**0.25**
DV: urge to eat			
Mindfulness^a^	−6.88 (−13.79; 0.04)	.051	−0.11
Self-compassion^a^	−2.49 (−9.44; 4.46)	.479	−0.04
DV: urge to purge			
Mindfulness^a^	−7.20 (−14.18; −0.22)	.043	−0.12
Self-compassion^a^	−5.70 (−12.67; 1.26)	.108	−0.09
DV: acceptance for shape and weight			
Mindfulness^a^	5.07 (−1.60; 11.75)	.135	0.11
Self-compassion^a^	4.42 (−2.13; 10.97)	.184	0.09
DV: feeling valuable			
Mindfulness^a^	5.72 (−2.13; 13.57)	.152	0.12
Self-compassion^a^	8.30 (0.68; 15.91)	.033	0.17

^a^Independent variables dummy-coded for comparison with cognitive restructuring and adjusted for baseline and pre-strategy values of the dependent variable

*95% CI* 95% confidence interval, *DV* dependent variable

Effects with *p* < .0125 in **boldface**

There was no statistically significant association between comorbid mental disorders and previous treatment, χ^2^(1) = 1.32, *p* = .251. Tentative evidence emerged that the advantage of contextual strategies over cognitive restructuring might be influenced by comorbid mental disorders and previous treatment experiences (Table 3). While there were no differences in patients without comorbidity, self-compassion outperformed cognitive restructuring in patients with comorbidity with regard to increasing perceived control over the present feeling. Again, there were no differences between ER strategies in patients without previous treatment. In patients with previous treatment, mindfulness outperformed cognitive restructuring in increasing acceptance for the present feeling and self-compassion outperformed cognitive restructuring with regard to improving perceived control over the present feeling.

**Table 3:** Influence of comorbid mental disorders and previous treatment on the adjusted efficacy of emotion regulation strategies: results of the multiple linear regression analyses

Variables	Comorbid mental disorders						Previous treatment					
	No			Yes			No			Yes		
	*b* (95% CI)	*p*	*β*	*b* (95% CI)	*p*	*β*	*b* (95% CI)	*p*	*β*	*b* (95% CI)	*p*	*β*
DV: sadness												
Mindfulness^a^	5.52 (−7.03; 18.06)	.380	0.10	−4.36 (−15.42; 6.70)	.435	−0.08	8.27 (−6.34; 22.88)	.258	0.15	−4.38 (−14.96; 6.20)	.413	−0.08
Self-compassion^a^	0.42 (−12.11; 12.96)	.068	0.01	−5.05 (−16.11; 6.02	.368	−0.10	3.02 (−11.62; 17.65)	.678	0.01	−5.39 (−16.03; 5.24)	.317	−0.10
DV: acceptance for feeling												
Mindfulness^a^	10.13 (−4.59; 24.84)	.171	0.20	11.11 (0.93; 21.29)	.033	0.23	11.88 (−7.11; 30.87)	.211	0.28	9.56 (0.32; 18.80)	.043	0.19
Self-compassion^a^	8.42 (−6.49; 23.32)	.260	0.17	8.30 (−1.73; 18.34)	.104	0.17	8.65 (−9.28; 26.58)	.331	0.20	7.84 (−1.44; 17.12)	.097	0.15
DV: tolerance for feeling												
Mindfulness^a^	7.36 (− 9.77; 24.50)	.389	0.15	8.87 (−2.09; 19.83)	.111	0.16	17.57 (−0.04; 35.18)	.050	0.41	4.87 (−5.79; 15.52)	.366	0.09
Self-compassion^a^	8.61 (−8.84; 26.05)	.323	0.17	11.38 (0.41; 22.35)	.042	0.21	16.02 (−2.10; 34.13)	.081	0.37	8.20 (−2.50; 18.91)	.131	0.23
DV: control feeling												
Mindfulness^a^	−1.02 (−13.14; 11.11)	.866	−0.03	6.40 (−2.58; 15.39)	.160	0.13	−2.71 (−16.18; 10.75)	.683	−0.08	4.79 (−3.52; 13.10)	.255	0.10
Self-compassion^a^	7.94 (−4.27; 20.14)	.195	0.19	**15.01 (5.75; 24.28)**	**.002**	**0.31**	3.69 (−10.05; 17.44)	.587	0.12	**13.68 (5.25; 22.11)**	**.002**	**0.27**
DV: urge to eat												
Mindfulness^a^	−14.03 (−28.19; 0.13)	.052	−0.25	−3.67 (−11.65; 4.30)	.362	−0.06	−12.37 (−28.26; 3.52)	.122	−0.17	−5.00 (−12.65; 2.74)	.204	−0.09
Self-compassion^a^	−6.97 (−21.41; 7.48)	.334	−0.12	−0.21 (−8.18; 7.76)	.958	−0.003	−14.93 (−30.84; 0.98)	.065	−0.20	1.89 (−5.88; 9.65)	.630	0.03
DV: urge to purge												
Mindfulness^a^	−5.20 (−15.89; 5.49)	**.**330	−0.09	−8.18 (−17.19; 0.83)	.075	−0.13	−5.03 (−17.46; 7.41)	.415	−0.08	−8.04 (−16.61; 0.54)	.066	−0.13
Self-compassion^a^	2.27 (−8.52; 13.05)	.672	0.04	−9.47 (−18.43; −0.52)	.038	−0.15	−2.27 (−14.66; 10.11)	.710	−0.04	−7.00 (−15.57; 1.56)	.108	−0.11
DV: acceptance for shape and weight												
Mindfulness^a^	−0.27 (−12.98; 12.45)	.966	−0.01	6.96 (−0.74; 14.65)	.076	0.15	−1.35 (− 12.83; 10.13)	.812	−0.03	7.54 (−0.71; 15.80)	.073	0.15
Self-compassion^a^	2.64 (−9.64; 14.92)	.665	0.06	5.38 (−2.20; 12.95)	.162	0.12	−6.86 (−18.27; 4.55)	.228	−0.17	8.52 (0.48; 16.56)	.038	0.17
DV: feeling valuable												
Mindfulness^a^	0.56 (−12.34; 13.45)	.931	0.01	7.38 (−2.67; 17.43)	.148	0.16	5.84 (−7.83; 19.52)	.389	0.14	5.04 (−4.44; 14.51)	.294	0.10
Self-compassion^a^	6.32 (−6.40; 19.05)	.320	0.14	8.76 (−0.90; 18.41)	.075	0.19	− 0.76 (−13.90; 12.39)	.907	−0.02	10.45 (1.22; 19.79)	.027	0.20

^a^Independent variables dummy-coded for comparison with cognitive restructuring and adjusted for baseline and pre-strategy values of the dependent variable

*95% CI* 95% confidence interval, *DV* dependent variable

Effects with *p* < .0125 in **boldface**

## Discussion

Applying an experimental paradigm, we examined the efficacy of cognitive restructuring, mindfulness, and self-compassion on emotional and ED-related outcomes after an unpleasant mood induction in patients with BN. With regard to our study aims, we found that (1) for three ED-related outcomes, contextual ER strategies were more efficacious than waiting. Cognitive restructuring did not differ from waiting for any outcome. (2) Contextual strategies were more efficacious than cognitive restructuring for two outcomes related to emotions, for the rest, there was no difference. (3) Self-compassion was more efficacious than cognitive restructuring with regard to perceived control over the current feeling in patients with comorbid mental disorders and previous treatment. Given the small sample size, our findings need to be considered preliminary.

Overall, self-compassion seemed to be the most efficacious ER strategy both in comparison to other strategies and in the sense of affecting multiple aspects of ER. This finding undergirds preliminary clinical evidence that introducing self-compassion to ED treatment improves outcome [38], emphasizes that self-compassion might advance the treatment of EDs [19], and concurs with findings from clinical [43], non-clinical [44], and experimental [45, 46] studies.

Contextual strategies showed associations with outcomes both related to emotions and ED-symptoms, including an increased perceived control over the feeling, which would rather be expected to result from cognitive restructuring. This observation is especially interesting as the instruction for self-compassion did not invite the patients to attempt any changes at their current emotions and impulses. Self-compassion also actually reduced the feeling of sadness to a larger extent than the other strategies.

The comparatively low efficacy of cognitive restructuring in our study is inconsistent with theoretical assumptions and empirical findings (e.g., [46, 47]). It is possible that a common side-effect of cognitive restructuring took effect, with some patients misunderstanding the instructions as implying that they have wrong thoughts and just need to change them [48]. Possibly, the design of the current study may be biased in producing better results for the contextual strategies. First, mindfulness and self-compassion interventions might be more easily delivered through a “one-way” format with a passively listening patient, while cognitive restructuring relies on debating thoughts and assumptions with an active therapist. Our efforts to recreate this scenario by including several questions in the instructions might have been too challenging for the participants. Second, cognitive restructuring seems to be difficult to apply during strong unpleasant emotional activation and rather affords a calm mind to be effective [49]. Third, cognitive restructuring may take more time to learn and would possibly show effects in a longitudinal study. One the one hand, these notions highlight that cognitive restructuring might be less easily examined in an experimental setting, on the other hand, they suggest that contextual strategies are more efficacious across outcomes and unfold their effect independently of factors related to the patient and the their method of delivery. For the correct interpretation of these findings, it also has to be acknowledged that the mood induction before cognitive restructuring may have been less effective than for the other conditions, making it less likely for an intervention to show an effect.

Our findings give tentative evidence that differences between the active ER strategies only emerged in patients with higher loads of psychopathology and previous treatment for BN. Applying a more strict control for multiple comparisons, self-compassion showed the only difference to cognitive restructuring with regard to perceived control over the current feeling in both patients with previous treatment and comorbid mental disorders. Inspecting the other trends suggested by the regression coefficients, self-compassion and mindfulness also outperformed cognitive restructuring in patients with comorbid mental disorders with regard to both emotional and ED-related outcomes. The former outcomes related to basic treatment elements of psychotherapy for BN, including emotional competence and disorder-specific elements. Accepting one’s body and weight and feeling valuable, however, are advanced outcomes for persons with EDs, as low self-esteem and body dissatisfaction are thought to be at the core of EDs [50]. Potentially, the patients with previous treatment were able to access their knowledge and experiences from these treatments when applying self-compassion and, thereby, able to produce larger gains during the experiment.

### Clinical implications

Our findings suggest that the treatment of BN might benefit from increasingly incorporating contextual strategies for ER. Especially self-compassion showed advantages over waiting and cognitive restructuring across a range of outcomes. Respective approaches have been formulated for EDs [19] and proven their efficacy for different disorders [43]. In contrast to cognitive restructuring, contextual strategies are easily applied in the daily life of the patients, for example, in the form of audio recordings that can be played on the way to work or at home. By that, they increase the patients’ possibilities to self-manage and take responsibility. Since ER deficits are a shared feature of most, if not all, mental disoders [1], these findings likely also apply to other mental disorders.

### Strengths and limitations

The strengths of the present study are the experimental design, the application of a waiting condition, and the standardized diagnostic assessment. Limitations of the study are the exclusively female and adult sample as well as the exclusive use of self-assessment. In the face of increased internal validity, the experimental setting precludes the generalization of our findings. Also, we are not able to make statements about long-term effects of the interventions and the lack of a clinical control group precludes any statements about the specificity of our findings to BN. Our sample size might have been to small to provide sufficient power to detect effects with regard to the number of tests performed. Our findings, therefore, have to bee seen as preliminary and require replication in larger samples. Ideally, these studies would examine further confounders, including previous experience with ER strategies and inter-individual differences in ER competency. While we were able to show that the permutation design did not create any order effects in post-strategy ratings, future studies should include a completely passive waiting group to better map possible intra-individual interactions between the ER strategies.

## Conclusions

Our findings suggest that contextual strategies, especially self-compassion, are more efficacious than waiting and cognitive restructuring in improving short-term ER in patients with BN in an experimental setting. These findings need to be transferred to applied settings.

## Data Availability

The dataset supporting the conclusion of this article are held by the authors and will be made available upon justified request.

## Abbreviations

ACT: Acceptance and commitment therapy
BN: Bulimia nervosa
CBT: Cognitive behavioural therapy
CFT: Compassion focused therapy
ED: Eating disorder
ER: Emotion regulation
MSC: Mindfulness self compassion training

## References

[1] Fernandez KC, Jazaieri H, Gross JJ (2016). Emotion regulation: a transdiagnostic perspective on a new RDoC domain. Cognit Ther Res.

[2] Svaldi J, Griepenstroh J, Tuschen-Caffier B, Ehring T (2012). Emotion regulation deficits in eating disorders: a marker of eating pathology or general psychopathology?. Psychiatry Res.

[3] Harrison A, Sullivan S, Tchanturia K, Treasure J (2010). Emotional functioning in eating disorders: attentional bias, emotion recognition and emotion regulation. Psychol Med.

[4] Sim L, Zeman J (2010). Emotion awareness and identification skills in adolescent girls with bulimia nervosa. J Clin Child Adolesc Psychol.

[5] Evers C, Stok F, de Ridder DTD (2010). Feeding your feelings: emotion regulation strategies and emotional eating. Personal Soc Psychol Bull.

[6] Smyth JM, Wonderlich SA, Heron KE, Sliwinski MJ, Crosby RD, Mitchell JE (2007). Daily and momentary mood and stress are associated with binge eating and vomiting in bulimia nervosa patients in the natural environment. J Consult Clin Psychol.

[7] Meule A, Richard A, Schnepper R, Reichenberger J, Georgii C, Naab S, et al. Emotion regulation and emotional eating in anorexia nervosa and bulimia nervosa. Eat Disord. 2019:1–17. 10.1080/10640266.2019.1642036 31345125

[8] Agras WS, Telch CF (1998). The effects of caloric deprivation and negative affect on binge eating obese binge-eating disordered women. Behav Ther.

[9] Chua JL, Touyz S, Hill AJ (2004). Negative mood-induced overeating in obese binge eaters: an experimental study. Int J Obes.

[10] Dingemans AE, Martijn C, Jansen ATM, van Furth EF (2009). The effect of suppressing negative emotions on eating behavior in binge eating disorder. Appetite..

[11] Kisler VA, Corcoran KJ (1997). Effects of negative outcome on food consumption in college women with and without troubled eating patterns. Addict Behav.

[12] Tuschen-Caffier B, Vögele C (1999). Psychological and physiological reactivity to stress: an experimental study on bulimic patients, restrained eaters and controls. Psychother Psychosom.

[13] MacDonald DE, Trottier K, Olmsted MP (2017). Rapid improvements in emotion regulation predict intensive treatment outcome for patients with bulimia nervosa and purging disorder. Int J Eat Disord.

[14] MacDonald DE, Trottier K (2019). Rapid improvements in emotion regulation predict eating disorder psychopathology and functional impairment at 6-month follow-up in individuals with bulimia nervosa and purging disorder. Int J Eat Disord.

[15] Peterson CB, Berg KC, Crosby RD, Lavender JM, Accurso EC, Ciao AC (2017). The effects of psychotherapy treatment on outcome in bulimia nervosa: examining indirect effects through emotion regulation, self-directed behavior, and self-discrepancy within the mediation model. Int J Eat Disord.

[16] Fairburn CG (2008). Cognitive behavior therapy and eating disorders.

[17] Wilson GT, Grilo CM, Vitousek KM (2007). Psychological treatment of eating disorders. Am Psychol.

[18] Prefit AB, Cândea DM, Szentagotai-Tătar A (2019). Emotion regulation across eating pathology: a meta-analysis. Appetite..

[19] Goss K, Allan S (2010). Compassion focused therapy for eating disorders eating disorder service. Int J Cogn Ther.

[20] Germer CK, Neff KD (2013). Self-compassion in clinical practice. J Clin Psychol.

[21] Hayes SC, Strosahl KD, Wilson KG (2016). Acceptance and commitment therapy: the process and practice of mindful change.

[22] Kabat-Zinn J (1994). Wherever you go, there you are: mindfulness meditation in everyday life.

[23] Butryn ML, Juarascio A, Shaw J, Kerrigan SG, Clark V, O’Planick A (2013). Mindfulness and its relationship with eating disorders symptomatology in women receiving residential treatment. Eat Behav.

[24] Masuda A, Wendell JW (2010). Mindfulness mediates the relation between disordered eating-related cognitions and psychological distress. Eat Behav.

[25] Anderson DA, Simmons AM (2008). A pilot study of a functional contextual treatment for bulimia nervosa. Cogn Behav Pract.

[26] Baer RA, Fischer S, Huss DB (2005). Mindfulness and acceptance in the treatment of disordered eating. J Ration Emotive Cogn Behav Ther.

[27] Kristeller JL, Baer RA, Quillian-Wolever R, Baer RA (2006). Mindfulness-based approaches to eating disorders. Mindfulness accept Interv conceptualization, Appl Empir Support.

[28] Linehan MM (1993). Cognitive-behavioral treatment of borderline personality disorder.

[29] Linehan MM (1993). Skills training manual for treating borderline personality disorder.

[30] Proulx K (2008). Experiences of women with bulimia nervosa in a mindfulness-based eating disorder treatment group. Eat Disord.

[31] Weissman S, Weissman R (1996). Meditation, compassion & loving kindness: an approach to vipassana practice.

[32] Swanepoel CR. A correlation study of self-compassion, self-forgiveness and eating disorder behaviour among university females. Potchefstroom: North-West University; 2009.

[33] Wasylkiw L, MacKinnon AL, MacLellan AM (2012). Exploring the link between self-compassion and body image in university women. Body Image.

[34] Ferreira C, Pinto-Gouveia J, Duarte C (2013). Self-compassion in the face of shame and body image dissatisfaction: implications for eating disorders. Eat Behav.

[35] Fennig S, Brunstein Klomek A, Shahar B, Sarel-Michnik Z, Hadas A (2017). Inpatient treatment has no impact on the core thoughts and perceptions in adolescents with anorexia nervosa. Early Interv Psychiatry.

[36] Speranza M, Atger F, Corcos M, Loas G, Guilbaud O, Stéphan P (2003). Depressive psychopathology and adverse childhood experiences in eating disorders. Eur Psychiatry.

[37] Kelly AC, Carter JC, Zuroff DC, Borairi S (2013). Self-compassion and fear of self-compassion interact to predict response to eating disorders treatment: a preliminary investigation. Psychother Res.

[38] Gale C, Gilbert P, Read N, Goss K (2014). An evaluation of the impact of introducing compassion focused therapy to a standard treatment programme for people with eating disorders. Clin Psychol Psychother.

[39] Wittchen HU, Zaudig M, Fydrich T (1997). Strukturiertes Klinisches Interview für DSM–IV.

[40] Velten EJ (1968). A laboratory task for induction of mood states. Behav Res Ther.

[41] Gerrards-Hesse A, Spies K, Hesse FW (1994). Experimental inductions of emotional states and their effectiveness: a review. Br J Psychol.

[42] Westermann R, Spies K, Stahl G, Hesse FW (1996). Relative effectiveness and validity of mood induction procedures: a meta- analysis. Eur J Soc Psychol.

[43] Wilson AC, Mackintosh K, Power K, Chan SWY (2019). Effectiveness of self-compassion related therapies: a systematic review and meta-analysis. mindfulness (N Y). Mindfulness.

[44] MacBeth A, Gumley A (2012). Exploring compassion: a meta-analysis of the association between self-compassion and psychopathology. Clin Psychol Rev.

[45] Diedrich A, Grant M, Hofmann SG, Hiller W, Berking M (2014). Self-compassion as an emotion regulation strategy in major depressive disorder. Behav Res Ther.

[46] Odou N, Brinker J (2015). Self-compassion, a better alternative to rumination than distraction as a response to negative mood. J Posit Psychol.

[47] Aldao A (2013). The future of emotion regulation research: capturing context. Perspect Psychol Sci.

[48] Hofmann SG, Asmundson GJG, Beck AT (2013). The science of cognitive therapy. Behav Ther.

[49] Sheppes G, Scheibe S, Suri G, Gross JJ (2011). Emotion-regulation choice. Psychol Sci.

[50] Fairburn CG, Cooper Z, Shafran R (2003). Cognitive behaviour therapy for eating disorders: a “ transdiagnostic ” theory and treatment. Behav Res Ther.

